# The second Symptom Management Research Trial in Oncology (SMaRT Oncology-2): a randomised trial to determine the effectiveness and cost-effectiveness of adding a complex intervention for major depressive disorder to usual care for cancer patients

**DOI:** 10.1186/1745-6215-10-18

**Published:** 2009-03-30

**Authors:** Jane Walker, Jim Cassidy, Michael Sharpe, the SMaRT Oncology-2 Trialists*

**Affiliations:** 1 Cancer Research UK Clinical Research Training Fellow, University of Edinburgh Cancer Research Centre, Western General Hospital, Crewe Road, Edinburgh, EH4 2XR, Scotland; 2 Professor of Oncology, Cancer Research UK, Department of Medical Oncology, University of Glasgow, Garscube Estate, Switchback Road, Glasgow, G61 1BD, Scotland; 3 Professor of Psychological Medicine Psychological Medicine Research School of Molecular and Clinical Medicine, University of Edinburgh, Kennedy Tower, Royal Edinburgh Hospital, Morningside Park, Edinburgh, EH10 5HF, Scotland

## Abstract

**Background:**

*Depression Care for People with Cancer *is a complex intervention delivered by specially trained cancer nurses, under the supervision of a psychiatrist. It is given as a supplement to the usual care for depression, which patients receive from their general practitioner and cancer service. In a 'proof of concept' trial (Symptom Management Research Trials in Oncology-1) *Depression Care for People with Cancer *improved depression more than usual care alone. The second Symptom Management Research Trial in Oncology (SMaRT Oncology-2 Trial) will test its effectiveness and cost-effectiveness in a 'real world' setting.

**Design:**

A two arm parallel group multi-centre randomised controlled trial.

**Trial Procedures:**

500 patients will be recruited through established systematic Symptom Monitoring Services, which screen patients for depression. Patients will have: a diagnosis of cancer (of various types); an estimated life expectancy of twelve months or more and a diagnosis of Major Depressive Disorder. Patients will be randomised to usual care or usual care plus *Depression Care for People with Cancer*. Randomisation will be carried out by telephoning a secure computerised central randomisation system or by using a secure web interface. The primary outcome measure is 'treatment response' measured at 24 week outcome data collection. 'Treatment response' will be defined as a reduction of 50% or more in the patient's baseline depression score, measured using the 20-item Symptom Checklist (SCL-20D). Secondary outcomes include remission of major depressive disorder, depression severity and patients' self-rated improvement of depression.

**Trial Registration:**

Current controlled trials ISRCTN40568538

**Trial Hypotheses:**

(1) *Depression Care for People with Cancer *as a supplement to usual care will be more effective than usual care alone in achieving a 50% reduction in baseline SCL-20D score at 24 weeks. (2) *Depression Care for People with Cancer *as a supplement to usual care will cost more than usual care alone but will be more cost effective in achieving improvements in patients' depression and quality of life.

## Background

When depression complicates medical conditions, it is associated with substantially reduced quality of life [1]. Patients also experience increased symptom burden and greater disability, and are less likely to adhere to medical treatments [2]. Although depression is common in patients who have cancer, it is frequently undetected and untreated [3].

*Depression Care for People with Cancer *is a complex intervention delivered by specially trained Care Managers (for the SMaRT Oncology-2 Trial these will be cancer nurses) supervised by a psychiatrist, which will supplement usual care. It is based on the 'collaborative care' model of depression management developed by the Seattle group [4-6], adapted for patients with cancer and a specialist setting.

Recent meta-analyses have found that interventions based on the 'collaborative care' model were more effective in improving depression outcomes than standard care for patients in primary care [7,8]. However, few trials have specifically included patients with co-morbid medical illnesses. Trials in patients with diabetes [9] and in older patients with various co-morbidities [6] have reported modest effects. A pilot study of collaborative care in low-income Latinas with co-morbid depression and cancer reported promising initial results [10].

*Depression Care for People with Cancer *has been found to be feasible to deliver [11] and the SMaRT oncology-1 'proof-of-concept' trial found that patients who received the intervention had significantly better outcomes than those who received usual care alone at three months (standardised effect size of 0.43) and that this difference was sustained at six and twelve months [12]. We now propose to evaluate the effectiveness and cost-effectiveness of this complex intervention in a large pragmatic multi-centre randomised controlled trial.

## Methods

### Design

A two-arm parallel group, multi-centre, randomised controlled trial with outcome data collection every 12 weeks to 48 weeks post-randomisation.

### Patients

500 patients will be recruited from selected oncology outpatient clinics in NHS Lothian and NHS Greater Glasgow and Clyde.

To be included in the trial patients must:

• Have a diagnosis of cancer, with active disease within the last five years.

• Be aged 18 or over.

• Be attending a specialist oncology clinic.

• Have a predicted survival, estimated by their cancer specialist, of twelve months or more.

• Have symptoms which meet DSMIV criteria for Major Depressive Disorder (MDD), using the inclusive approach to diagnosis with the current Major Depressive Episode (MDE) having being present for four weeks or more.

Patients will be excluded if:

• They are unable to provide informed consent to participate.

• The episode of depression is chronic (defined as a history of continuous depression for at least two years).

• They are judged to require urgent psychiatric care.

• They are receiving active psychiatric or psychological treatment from specialist mental health services.

• They have cognitive impairment or communication difficulties (including inability to adequately understand verbal explanations or written information in English) which are incompatible with the intervention.

• They have known cerebral metastases.

• They are unable to attend regularly for treatment sessions.

• The intervention is judged to be inappropriate due to a medical condition which requires alternative treatment.

• The intervention is judged to be inappropriate due to a psychiatric condition which requires alternative treatment (psychotic illness, bipolar affective disorder, obsessive compulsive disorder, substance abuse or dependence).

• Their participation in the trial is judged to be inappropriate on other clinical grounds.

N.B Patients receiving active cancer treatments will not be excluded unless they fulfil one or more of the exclusion criteria listed above.

### Patient identification and enrolment

In order to obtain a representative sample, patients will be identified through the Symptom Monitoring Service (SMS) in selected oncology outpatient clinics of NHS Lothian and NHS Greater Glasgow and Clyde.

The SMS is part of the routine NHS care for patients attending these clinics. It comprises two stages:

• Stage 1 Patients complete self-report questionnaires in the clinic to evaluate their symptoms, including emotional distress (Hospital Anxiety and Depression Scale, HADS). A summary of each patient's symptoms is generated for their oncology team.

• Stage 2 Patients with high emotional distress scores (HADS total score of 15 or more) receive a brief telephone-delivered, depression screening interview administered by trained staff (graduate research assistants). The interview comprises the major depression component of the Structured Clinical Interview for DSMIV (SCID). When clinically relevant, a report is generated for the patient's general practitioner (GP) and oncologist. The SMS staff will ask patients, with an MDE of at least four weeks duration and a cancer prognosis of twelve months or more, to give permission for the research team to contact them. It will be made clear that patients who do so will not be obliged to participate in the trial.

Once patients have given permission, the research team provide them with a trial information leaflet (usually by post) and will contact them directly (usually by telephone). Patients will be informed, during the telephone call, that if they are interested in taking part, their suitability for inclusion in the trial must first be assessed. Information will be required from patients' medical records in order to achieve this.

Patients will therefore be asked, at this stage, to give verbal consent for the research team to access their medical records. This will minimise the burden on patients of unnecessary face-to-face assessments. Consent to access medical records is required as the researchers do not provide the patient's usual clinical care. The patient's records will be used to determine the presence or absence of specific inclusion and exclusion criteria, before they are seen for a face-to-face assessment (if appropriate). If the patient does not give consent for the research team to access their records at this stage, consent for this will be obtained during the face-to-face assessment.

At the face-to-face assessment, patients will be given a full explanation of the two treatment conditions and of the procedures for randomised allocation to these and for outcome data collection. Written informed consent will then be obtained from patients for the eligibility assessment and, if appropriate, trial participation. Patients will be asked to consent to the information gathered about them being retained even if they are found to be ineligible to participate in the trial. This information will be used to inform an estimate of the generalisability of the trial findings.

The information retained from medical records and the face-to-face assessment will be used to determine eligibility. Eligibility assessments will be carried out by trained research staff (senior nurses, psychologists and psychiatrists) and will include administration of the major depression component of the Structured Clinical Interview for DSMIV (SCID) to confirm the patient's diagnosis of major depression. If the patient is eligible to participate, their willingness to do so will be confirmed before randomisation. The patient's details will be entered into a database via an automated telephone service or secure website. The patient's treatment allocation will be automatically generated. The research team will inform the patient of their allocation either face-to-face or by telephone.

At all stages the research team will endeavour to record reasons for non-participation.

### Trial treatment – comparison (usual care)

The patient's GP and oncologist will be informed of their diagnosis of MDD. Patients will receive the usual clinical management for their depression. Data will be collected from all patients to allow a retrospective description of the 'usual care' that they receive.

### Trial treatment – intervention (usual care supplemented with *Depression Care for People with Cancer*)

In addition to the usual care described above, patients allocated to the condition will receive *Depression Care for People with Cancer*. This will be delivered by specially trained Care Managers (cancer nurses) under the supervision of the SMaRT Psychiatry team. Patients will be allocated to Care Managers systematically, by being allocated to the next Care Manager on the appropriate site-specific list. If the allocated Care Manager is unable to see the patient for their first treatment session within two weeks (e.g. due to leave or staff sickness), the patient will be allocated to the next listed Care Manager.

The intervention is described in detail in the *Depression Care for People with Cancer Treatment Manual *and comprises two phases:

(a) Treatment Phase: patients will be offered a maximum of ten 30–45 minute sessions with their Care Manager over a 16 week period (patients are expected to receive an average of six to eight sessions).

(b) Maintenance phase: patients will then receive active treatment follow-up by telephone every four weeks until the end of their participation in the trial.

During the Treatment Phase the patient's Care Manager will: (a) coordinate their depression care by liaising with all relevant health professionals; (b) monitor their symptoms of depression, using a brief standardised depression questionnaire (Patient Health Questionnaire-9, PHQ-9) at each session; (c) provide a brief psychological intervention comprising education about depression and its management (including the use of antidepressant medication, being active and coping with problems better) and Problem Solving Treatment. Where possible, treatment sessions will be delivered at the cancer clinics of NHS Lothian and NHS Greater Glasgow and Clyde. Treatment sessions will be delivered at home or by telephone for patients who are unable to attend the hospital.

During the Maintenance Phase patients will be asked to complete the PHQ-9 at monthly follow-up telephone calls. Patients' responses to this questionnaire will be reviewed by their Care Manager and appropriate action will be taken as necessary. Follow-up telephone calls will be administered by an Interactive Voice Response (IVR) computerised telephone system or by the Care Manager for patients who are unable or unwilling to use the system. Patients will be given a full explanation of the follow-up procedure (including how to use the IVR system when appropriate) during the Treatment Phase.

All patients will be discussed during weekly supervision sessions for Care Managers, provided by the SMaRT Psychiatry Team. In addition, a member of the Psychiatry Team will be available to respond to emergencies. At any stage of treatment patients who report suicidal thoughts will be discussed with the Psychiatry Team and an appropriate management plan implemented. A supervising psychiatrist will review all patients who in their opinion: (a) require a diagnostic re-assessment; (b) have failed to achieve a treatment response (defined as a 50% drop in their PHQ-9 score *and *a PHQ-9 score of <10) by session five or week eight of the Treatment Phase (whichever is earlier); (c) require assessment for referral to general adult psychiatric care (patients with high suicide risk, psychosis or mania and those requiring inpatient care).

Patients' adherence to the intervention will be monitored by recording their attendance at treatment sessions, completion of homework, antidepressant prescription (drug and dose) and self-reported compliance with medication.

In order to ensure Care Manager's compliance with the treatment manual, all treatment sessions (of patients who consent to this) will be digitally video or audio recorded. Recordings and treatment notes will be reviewed regularly by the SMaRT Psychiatry Team and a randomly selected 10% sample of recordings will be independently reviewed and rated on a standardised adherence checklist, designed specifically for this intervention.

### Primary outcome measure

The primary outcome measure is 'treatment response' measured at 24 week outcome data collection. 'Treatment response' will be defined as a reduction of 50% or more in the patient's baseline SCL-20D score. A binary primary outcome was chosen as this is a pragmatic trial and an outcome of clear clinical relevance is therefore required.

### Secondary outcome measures

The following secondary outcome measures will be assessed:

• Remission of major depressive disorder, defined as an SCL-20D score of < 0.75 at 24 and 36 and 48 weeks (higher than in primary care trials, to allow for cancer-related somatic symptoms).

• Depression severity, defined for each patient as the average of their SCL-20D score at 24, 36 and 48 weeks.

• Patient's self-rated improvement of depression, measured by a five point global improvement scale.

### Tertiary outcome measures

The following tertiary outcome measures will be assessed at 12, 24, 36, and 48 weeks from randomisation:

• Severity of depression symptoms, measured by the SCL-20D.

• Remission of major depressive disorder, defined as an SCL-20D score of < 0.75.

• Remission of major depressive disorder, defined as an SCL-20D score of < 0.5.

• Severity of anxiety symptoms, measured by the SCL-10A.

• Severity of pain and fatigue, measured by the relevant symptom scales of the EORTC QLQ-C30.

• Physical, social and role functioning, and overall health and quality of life measured by the relevant scales of the EORTC QLQ-C30.

• Patient's satisfaction with care, measured by a 5-point Likert scale item developed specifically for the trial.

### Measures of cost and health-related quality of life

The following economic outcome measures will be assessed at 12, 24, 36 and 48 weeks from randomisation:

• Cost of 'usual care' treatments received for depression.

• Cost of health care service use (primary, secondary and community based).

• Cost of the trial intervention.

• Health related quality of life, measured by EQ-5D.

### Data collection

Baseline data will be obtained by the assessor at the end of the face-to-face eligibility assessment as near as possible prior to randomisation

Outcome data collection will be obtained by a dedicated team who are blind to group allocation and are situated and managed independently from the trial team. This team will be managed by the Scottish Mental Health Research Network (SMHRN). Outcome data will be collected at 12, 24, 36 and 48 weeks from randomisation.

All baseline and outcome data will be collected using self-report questionnaires sent by mail with telephone calls or face-to-face interviews to collect missing items or whole questionnaires in order to maximise response rate. The date of completion of all questionnaires will be noted. Data collection will aim to be within one week either side of the specified follow-up date.

### Data collection instruments

The following data will be collected from patients at baseline, 12, 24, 36 and 48 weeks

• Symptom Checklist Depression Scale (SCL-20D)

• Symptom Checklist Anxiety Scale (SCL-10A)

• European Organisation for Research and Treatment of Cancer Quality of Life Questionnaire (EORTC-QLQ-C30)

• Patient's satisfaction with depression care (question developed specifically for the trial)

• EuroQol-5D (EQ-5D)

• Treatments received for depression (measures developed specifically for the trial)

• Use of health care services (measures developed specifically for the trial)

At baseline, demographic details and information about the patient's cancer will also be collected.

### Sample size

The power calculation is based on the primary outcome measure, namely 'treatment response' defined as a reduction of 50% or more in baseline SCL-20D score at 24 weeks. Two treatment groups of 240 patients (i.e. 480 in total) will give 90% power to detect an increase in the response rate from 35% to 50% at the 5% significance level, and 80% power to detect an increase in the response rate from 35% to 48% at the 5% significance level. Targeting recruitment at a total of 500 patients allows for a 4% loss to follow up at six months. This loss to follow-up rate is comparable with that achieved in the recently completed efficacy trial.

### Method of randomisation

In order to ensure similarity in the baseline characteristics of the two treatment groups, randomisation will be carried out using a combination of stratification and minimisation. Stratification will be by trial centre (Edinburgh, Glasgow) and minimisation variables will be age (≤50, 51–60, >60), cancer type (breast, genito-urinary, gynaecological, other) and sex. The minimisation variables are all equally weighted and will be balanced within each centre according to a deterministic algorithm. When the two treatment arms are perfectly balanced in terms of the age, cancer type and gender characteristics of a new subject, treatment allocation will occur randomly with equal probability assigned to the two treatments. The randomisation system is maintained by the Division of Clinical Neuroscience (DCN) Clinical Trials Unit at the University of Edinburgh, and allows for the real-time randomisation of patients using either a telephone (IVR) or web-based user interface.

### Statistical analyses

A single main analysis will be performed at the end of the trial when all outcome data have been collected. An independent Data Monitoring Committee will review confidential interim analyses of accumulating data at least annually. A detailed Statistical Analysis Plan will be developed prior to closure of the trial database and prior to the unblinding of the treatment allocations. The main analyses will be performed blind to treatment allocation on the 'intention-to-treat' principle, incorporating all randomised patients with usable outcome data.

The primary outcome measure of treatment response at 24 weeks will be analysed using logistic regression, with the minimisation variables being included as covariates. The results will be reported as an adjusted odds ratio with its corresponding 95% confidence interval.

The secondary outcome measure of remission of major depressive disorder at each and all of 24, 36 and 48 weeks will be analysed in the same way as the primary outcome measure.

The secondary outcome measure of depression severity averaged over 24, 36 and 48 weeks will be analysed using analysis of covariance, with the baseline SCL-20D score and the minimisation variables being included as covariates. The results will be reported as an adjusted mean difference in SCL-20D score with its corresponding 95% confidence interval.

### Economic analyses

The economic analyses will examine the cost-effectiveness of the complex intervention from an NHS perspective. Resource usage will be collected in the trial in relation to primary health care, community health and secondary health care services. These data will be collected from questionnaires administered to patients at each follow up point. The questionnaires will be designed for this study and will be based on questionnaires used in economic evaluations of other interventions.

Unit cost estimates, from routine published sources such as PSSRU Unit Costs of Health and Social Care [13], will then be applied to resource use data collected above to generate patient level cost estimates which will be presented as a mean cost with a measure of uncertainty. EQ-5D data will facilitate estimation of health-related quality of life, which will be expressed in terms of health states within the 245-state classification and in terms of health state values based on the preferences of a sample of the UK public [14]. Mean utility for each treatment allocation will be estimated with a measure of uncertainty. The analyses above will enable the estimation of mean differences in costs and effects for the treatment and control allocations. These will be presented with 95% confidence intervals around the differences in costs and effects. Uncertainty will be represented using the cost effectiveness acceptability curve (CEAC), a graphical representation of the probability of an intervention being cost-effective [15].

## Approvals and Sponsorship

SMaRT Oncology-2 has been approved, with favourable assessments at all sites (ref: 08/S11ADMIN/49; 08/S0701/63), by the Scotland A Research Ethics Committee (ref: 08/MRE00/23). Management approval has been granted by the Research and Development Offices of NHS Lothian and NHS Greater Glasgow.

The trial is co-sponsored by the University of Edinburgh and NHS Lothian Health Board.

## Timescales

Recruitment commenced May 2008. The target for recruitment closure is June 2010, with outcome data collection continuing until June 2011.

## Competing interests

The authors declare that they have no competing interests.

## Authors' contributions

JW participated in the design and coordination of the trial and drafted the manuscript. JC participated in the coordination of the trial. MS conceived of the trial, participated in its design and coordination and helped to draft the manuscript. All authors read and approved the final manuscript.

## Note

*Trial Management: Michael Sharpe, Jim Cassidy, Gordon Murray, David Weller, Jane Walker, Christian Hansen, Gerry Richardson, Lucy Wall, Mark Sculpher, Stephen Palmer.

Trial Steering Committee: Amanda Ramirez, Graham Dunn, Michael Bennett, Galina Velikova, Gillian McHugh, Simon Gilbody, Alison Richardson, Peter Rainey.

Data Monitoring Committee: John Geddes, Ed Juszczak, Dan Stark.
